# Chronic Loss of Melanin-Concentrating Hormone Affects Motivational Aspects of Feeding in the Rat

**DOI:** 10.1371/journal.pone.0019600

**Published:** 2011-05-05

**Authors:** Joram D. Mul, Susanne E. la Fleur, Pim W. Toonen, Anthonieke Afrasiab-Middelman, Rob Binnekade, Dustin Schetters, Michel M. M. Verheij, Robert M. Sears, Judith R. Homberg, Anton N. M. Schoffelmeer, Roger A. H. Adan, Ralph J. DiLeone, Taco J. De Vries, Edwin Cuppen

**Affiliations:** 1 Hubrecht Institute, Royal Netherlands Academy of Arts and Sciences and University Medical Center Utrecht, Utrecht, The Netherlands; 2 Department of Neuroscience and Pharmacology, Rudolf Magnus Institute of Neuroscience, University Medical Center Utrecht, Utrecht, The Netherlands; 3 Department of Cognitive Neuroscience, Donders Institute for Brain, Cognition, and Behavior, Radboud University, Nijmegen, The Netherlands; 4 Department of Molecular Animal Physiology, Donders Institute for Brain, Cognition, and Behavior, Radboud University, Nijmegen, The Netherlands; 5 Department of Anatomy and Neurosciences, Center for Neurogenomics and Cognitive Research, Free University Medical Center, Amsterdam, The Netherlands; 6 Department of Psychiatry, Ribicoff Research Facilities, Yale University School of Medicine, New Haven, Connecticut, United States of America; 7 Department of Neurobiology, Yale University School of Medicine, New Haven, Connecticut, United States of America; 8 Department of Medical Genetics, University Medical Center Utrecht, Utrecht, The Netherlands; University of Texas Health Science Center at San Antonio, United States of America

## Abstract

Current epidemic obesity levels apply great medical and financial pressure to the strenuous economy of obesity-prone cultures, and neuropeptides involved in body weight regulation are regarded as attractive targets for a possible treatment of obesity in humans. The lateral hypothalamus and the nucleus accumbens shell (AcbSh) form a hypothalamic-limbic neuropeptide feeding circuit mediated by Melanin-Concentrating Hormone (MCH). MCH promotes feeding behavior via MCH receptor-1 (MCH1R) in the AcbSh, although this relationship has not been fully characterized. Given the AcbSh mediates reinforcing properties of food, we hypothesized that MCH modulates motivational aspects of feeding.

Here we show that chronic loss of the rat MCH-precursor *Pmch* decreased food intake predominantly via a reduction in meal size during rat development and reduced high-fat food-reinforced operant responding in adult rats. Moreover, acute AcbSh administration of Neuropeptide-GE and Neuropeptide-EI (NEI), both additional neuropeptides derived from *Pmch*, or chronic intracerebroventricular infusion of NEI, did not affect feeding behavior in adult *pmch* ^+/+^ or *pmch* ^−/−^ rats. However, acute administration of MCH to the AcbSh of adult *pmch* ^−/−^ rats elevated feeding behavior towards wild type levels. Finally, adult *pmch* ^−/−^ rats showed increased ex vivo electrically evoked dopamine release and increased limbic dopamine transporter levels, indicating that chronic loss of *Pmch* in the rat affects the limbic dopamine system.

Our findings support the MCH-MCH1R system as an amplifier of consummatory behavior, confirming this system as a possible target for the treatment of obesity. We propose that MCH-mediated signaling in the AcbSh positively mediates motivational aspects of feeding behavior. Thereby it provides a crucial signal by which hypothalamic neural circuits control energy balance and guide limbic brain areas to enhance motivational or incentive-related aspects of food consumption.

## Introduction

The Melanin-Concentrating Hormone (MCH) precursor (*Pmch*) is predominantly expressed in neurons of the lateral hypothalamus (LHA) and the incerto hypothalamic area (sometimes referred to as zona incerta), which project throughout the brain [1], [2], [3]. *Pmch* processing generates glycine-glutamic acid (NGE), glutamic acid-isoleucine (NEI), and MCH [4]. MCH, a 19-amino acid cyclic peptide, is a key regulator of food intake and metabolism; *Pmch* mRNA is upregulated after fasting [5], [6], *Pmch*-deficient (*pmch* ^−/−^) rodents are hypophagic, lean, and have a decreased body weight as compared to wild type siblings [7], [8], [9], whereas *Pmch* overexpression results in hyperphagia and obesity [10]. Finally, intracerebroventricular (ICV) administration of MCH increases feeding [6], [11], [12], [13], [14], [15], [16].

In rodents MCH binds to MCH receptor-1 (MCH1R), which is present at high levels in limbic regions [17], [18], [19], [20], [21]. For example, the nucleus accumbens shell (AcbSh) modulates orexigenic activity of MCH [15], [21]. *Mch1r*-deficient mice are lean, hyperphagic, and hyperactive [22], [23], central blockade of MCH1R lowers body weight and food intake through several mechanisms [24], [25], [26], and acute central MCH1R-blockade decreases high-fat food-reinforced operant responding [27].

Dopamine is an important neurotransmitter in the nucleus accumbens (NAc), and absence of MCH-mediated signaling in mice increased NAc dopamine release and NAc dopamine receptor levels [28], [29], [30], [31]. MCH1R is expressed in AcbSh medium spiny neurons (MSNs) [21] and coexpressed with dopamine D_1_ and D_2_ receptors (D_1_R and D_2_R, respectively) [32]. Finally, reductions of AcbSh neuronal excitability, either by administration of MCH, an AMPA receptor antagonist, or a GABA receptor agonist all stimulate baseline-feeding behavior [33], [34], [35], [36], [37], [38], supporting a cellular excitability hypothesis of AcbSh-mediated feeding behavior [39].

The above findings link the hypothalamic orexigenic MCH circuit with neuronal AcbSh signaling activity that influences behavioral responses to rewarding stimuli including food and drugs of abuse. Furthermore, these findings have also generated interest from the pharmaceutical industry, as functional blockade of MCH1R in humans could be a potential target for the treatment of obesity.

In this study we use a recently generated rat knockout model [8] to investigate how chronic loss of *Pmch* in the rat affects motivational or incentive-related aspects of feeding behavior.

## Results

### Meal structure analysis in pmch^−/−^ rats during rat development

**Figure 1:**
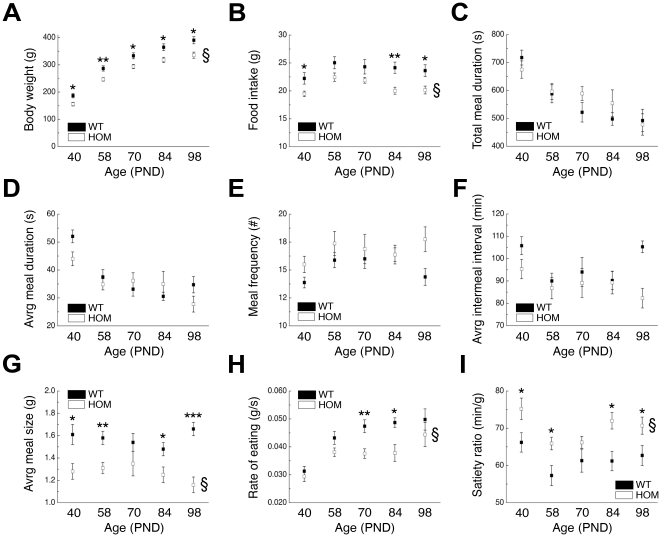
Meal structure analysis in *pmch* ^+/+^ and *pmch* ^−/−^ rats. (**A**) Body weight, (**B**) food intake, (**C**) total meal duration, (**D**) average meal duration, (**E**) meal frequency, (**F**) average intermeal interval, (**G**) average meal size, (**H**) rate of eating, and (**I**) satiety ratio in *pmch* ^+/+^ and *pmch* ^−/−^ rats at postnatal days [PND] 40, 58, 70, 84, and 98. Body weight, food intake, average meal size, and rate of eating are predominantly decreased in *pmch* ^−/−^ rats, while satiety ratios are predominantly increased in *pmch* ^−/−^ rats (§, *P*<0.05, WT vs. HOM; *P<0.05, **P<0.005, ***P<0.001, Students' *t*-test; n = 8 per group). Data are shown as mean ± S.E.M.

A previous study showed that chronic loss of *Pmch* in the rat resulted in hypophagia as compared to wild-type rats [8]. To investigate in more detail which elements of feeding behavior are changed during the observed hypophagia, we performed a meal structure analysis (parameters: body weight, food intake, total meal duration, average meal duration, meal frequency, average intermeal interval, average meal size, rate of eating, and satiety ratio) in *pmch* ^+/+^ and *pmch* ^−/−^ rats at PNDs 40, 58, 70, 84, and 98 (Figs. 1A – I).

The statistical analyses revealed a *genotype* effect for body weight, food intake, average meal size, rate of eating, and satiety ratio (Table S1). In addition, a *time* x *genotype* interaction was observed for body weight and average meal size (Table S1).

Although some parameters were affected at individual time points, robust effects after chronic loss of *Pmch* in the rat were only observed for body weight, food intake, and average meal size. Because average meal size is markedly affected, but meal duration is not, this translates to changes in rate of eating and satiety ratio.

### Decreased acute hyperphagia in pmch^−/−^ rats when newly presented with high fat diet

To investigate whether the hypophagia in *pmch* ^−/−^ rats (this study and [8]) results from perturbed food-induced reward signaling, we measured acute hyperphagia of adult *pmch* ^+/+^ and *pmch* ^−/−^ rats, grown on a standard semi-high protein (SHP) diet, when newly offered a palatable high-fat (HF) diet. In addition, acute hypophagia in response to newly offered SHP diet to adult rats grown on an HF diet was also investigated.

**Figure 2:**
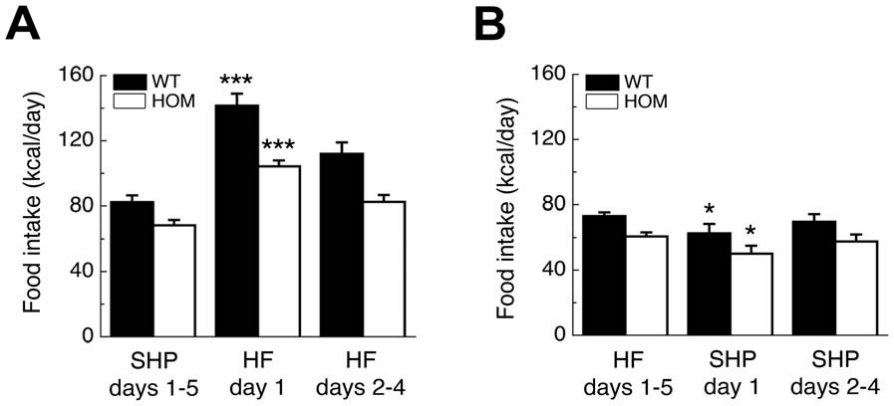
Acute hyperphagia in *pmch* ^−/−^ rats when newly presented with HF diet. (**A**) Adult *pmch* ^+/+^ and *pmch* ^−/−^ rats grown on standard SHP diet showed acute hyperphagia when newly presented with HF diet. However, acute hyperphagia was attenuated in *pmch* ^−/−^ rats (172±6% increase in *pmch* ^+/+^ rats compared to 152±6% increase in *pmch* ^−/−^ rats; ***P<0.001 versus SHP days1–5, repeated-measures ANOVA with special contrast analysis; n = 7–8 per group). (**B**) Adult *pmch* ^+/+^ and *pmch* ^−/−^ rats grown on HF diet showed acute hypophagia when newly presented with SHP diet. Acute hypophagia was equal between genotypes (14±8% decrease in *pmch* ^+/+^ rats as compared to 17±7% decrease in *pmch* ^−/−^ rats; *P<0.05 versus HF days1–5, repeated-measures ANOVA with special contrast analysis; n = 6–8 per group). Data are shown as mean ± S.E.M.

Home-cage food intake during basal days (days 1–5) was decreased in *pmch* ^−/−^ rats as compared to *pmch* ^+/+^ rats on both SHP and HF diet (*P*<0.01, by Student's *t*-test; Figs. 2A and B). Statistical comparison of baseline levels revealed a significant effect of *diet* (F_(1,25)_ = 12; *P*<0.005) and of *genotype* (F_(1,25)_ = 29; *P*<0.001), but no *diet* x *genotype* interaction (F_(1,25)_ = 0.07; *P* = 0.79). This suggests that *pmch* ^−/−^ rats respond equally to the intrinsic rewarding properties of the HF diet as compared to *pmch* ^+/+^ rats. However, statistical analysis for acute hyperphagia, when offered a HF diet for the first time, revealed a significant effect of *time* (F_(6,77)_ = 41; *P*<0.001), of *genotype* (F_(1,13)_ = 16; *P*<0.005), and a *time* x *genotype* interaction (F_(6,77)_ = 41; *P*<0.005; Fig. 2A). Although both *pmch* ^+/+^ and *pmch* ^−/−^ rats increased their caloric intake significantly during the first day on HF diet (F_(1,13)_ = 191.9; *P*<0.001), the increase in caloric intake showed an increased trend in *pmch* ^+/+^ rats as compared to *pmch* ^−/−^ rats (172±6% versus 152±6%, respectively; *P* = 0.060, Student's *t*-test).

When adult *pmch* ^+/+^ and *pmch* ^−/−^ rats, raised on an HF diet, were switched to SHP diet, the statistical analysis for acute hypophagia revealed significant effects of *time* (F_(4,46)_ = 5; *P*<0.005) and *genotype* (F_(1,12)_ = 14; *P*<0.005), but no *time* x *genotype* interaction (F_(4,77)_ = 0.5; *P* = 0.73; Fig. 2B). Both *pmch* ^+/+^ and *pmch* ^−/−^ rats reduced their caloric intake during the first day on SHP diet (F_(1,12)_ = 8; *P*<0.05). However, during acute hypophagia the decrease was equal between *pmch* ^+/+^ and *pmch* ^−/−^ rats (14±8% decrease versus 17±7% decrease, respectively; *P* = 0.74, Student's *t*-test).

### Loss of Pmch in the rat reduces high-fat food-reinforced operant responding

**Figure 3:**
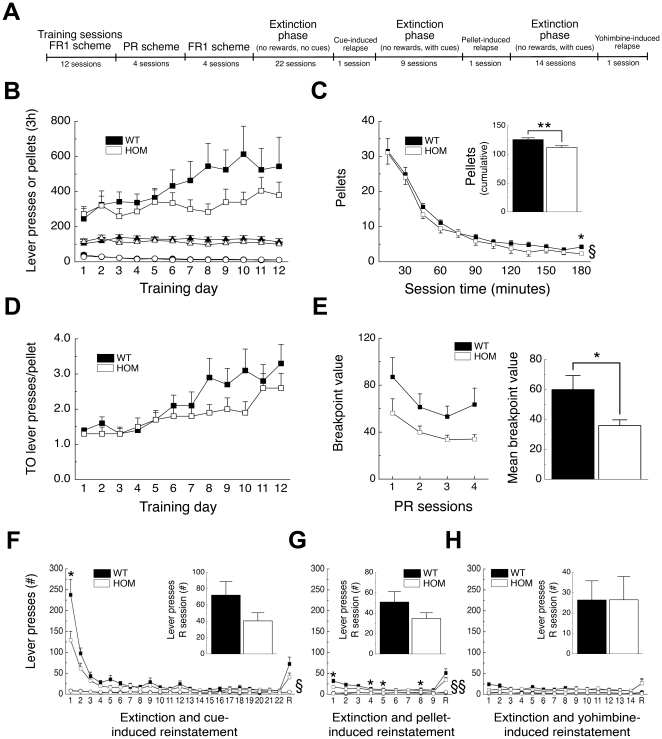
*Pmch* ^−/−^ rats show decreased HF food-reinforced operant responding for 45% fat pellets. (**A**) Experimental timeline for HF food-reinforced operant responding paradigm. (**B**) Number of total active-lever presses (squares), total inactive-lever presses (circles), and total number of pellets (triangles) during training sessions over 12 alternating days (one 3 hr session per day, every other day). (**C**) Mean pellets earned during 12 training sessions (total per 15-min bin time course) or per 3 hr (inset). (**D**) Total number of time-out (TO) presses per pellet during training sessions. (**E**) Breakpoint values of progressive ratio (PR) sessions over 4 alternating days (one 3 hr session per day, every other day), and mean breakpoint value of the last 3 PR sessions. Number of total active-lever presses (squares) and total inactive-lever presses (circles) during extinction sessions before (**F**) cue-induced (22 extinction sessions), (**G**) pellet-induced (9 extinction sessions), or (**H**) yohimbine-induced (14 extinction sessions) reinstatement (R, reinstatement session) of food seeking. Inset shows R session enlarged (n = 10 per group). §, *P*<0.05, §§, *P*<0.005, WT vs. HOM, repeated-measures ANOVA; *P<0.05, **P<0.005, Students' *t*-test. Data are shown as mean ± S.E.M.

As *pmch* ^−/−^ rats demonstrated hypophagia, we set out to investigate if *pmch* ^−/−^ rats have a decreased incentive, or motivation, for HF food-reinforced operant responding. Adult food-limited male *pmch* ^+/+^ and *pmch* ^−/−^ rats were tested in a self-administration paradigm where rats could lever press for 45% HF pellets (Fig. 3A).

During the training phase (FR1 schedule) rats were given 3 hr access to the pellets every other day. Statistical analysis for total active-lever presses during training revealed a significant effect of *time* (F_(4,71)_ = 4; *P*<0.05), but not of *genotype* (F_(1,18)_ = 1; *P* = 0.25) and a trend for the *time* x *genotype* interaction (F_(4,71)_ = 2; *P* = 0.08; Fig. 3B). The average number of pellets earned per training sessions was stable over time and statistical analysis revealed no significant effect of *time* (F_(7,128)_ = 1; *P* = 0.70) or of *genotype* (F_(1,18)_ = 1; *P* = 0.40), and also no *time* x *genotype* interaction (F_(7,128)_ = 1; *P* = 0.43; Fig. 3B). Statistical analysis for total inactive-lever presses revealed a significant effect of *time* (F_(5,84)_ = 11; *P*<0.001), but not of *genotype* (F_(1,18)_ = 0.2; *P* = 0.66) and no *time* x *genotype* interaction (F_(5,84)_ = 0.7; *P* = 0.61; Fig. 3B). Time-course analysis of the mean pellets earned within the training sessions revealed a significant effect of *time* (F_(2,37)_ = 93; *P*<0.001) and of *genotype* (F_(1,22)_ = 12; *P*<0.005), but no *time* x *genotype* interaction (F_(2,37)_ = 0.1; *P* = 0.84; Fig. 3C). Mean cumulative pellet intake per training session was decreased in *pmch* ^−/−^ rats as compared to *pmch* ^+/+^ rats (*P*<0.005, Student's *t*-test; Fig. 3C, *inset*). Timeout active-lever presses per pellet earned during training revealed a significant effect of *time* (F_(3,47)_ = 14; *P*<0.001), but not of *genotype* (F_(1,14)_ = 2; *P* = 0.24) and no *time* x *genotype* interaction (F_(3,47)_ = 3; *P* = 0.07; Fig. 3D). This observation mirrors the progressive escalation of timeout active lever presses across sessions (data not shown), which has been previously reported [27], [40], [41], [42], [43].

During the progressive ratio (PR) phase [44] rats were given 3 hr access to the pellets every other day. Statistical analysis for breakpoint values during PR revealed a significant effect of *time* (F_(3,54)_ = 8; *P*<0.001), a trend effect of *genotype* (F_(1,18)_ = 4; *P* = 0.052), but no *time* x *genotype* interaction (F_(3,54)_ = 0.5; *P* = 0.72; Fig. 3E). Mean cumulative breakpoint value of the last three sessions was decreased in *pmch* ^−/−^ rats as compared to *pmch* ^+/+^ rats (*P*<0.05, Student's *t*-test; Fig. 3E).

After PR, rats were tested on a FR1 schedule for 4 additional 3-hr sessions (data not shown) followed by 22 3-hr extinction sessions. Statistical analysis for total active-lever presses during these 22 extinction sessions revealed a significant effect of *time* (F_(21,378)_ = 46; *P*<0.001) and of *genotype* (F_(1,18)_ = 7; *P*<0.05), and a *time* x *genotype* interaction (F_(21,378)_ = 4; *P*<0.001; Fig. 3F). Cue-induced reinstatement did not differ between genotypes (*P* = 0.12; Student's *t*-test; Fig. 3F, *inset*). Nine additional extinction sessions preceded pellet-induced reinstatement. Statistical analysis for total active-lever presses during these 9 extinction sessions revealed a significant effect of *time* (F_(5,90)_ = 8; *P*<0.001) and of *genotype* (F_(1,18)_ = 14; *P*<0.005), but no *time* x *genotype* interaction (F_(5,90)_ = 1; *P* = 0.43; Fig. 3G). Pellet-induced reinstatement did not differ between genotypes (*P* = 0.12; Student's *t*-test; Fig. 3G, *inset*). Fourteen additional extinction sessions preceded yohimbine (an *α*_2_-adrenergic receptor antagonist and pharmacological stressor)-induced reinstatement. Statistical analysis for total active-lever presses during these 14 extinction sessions revealed no significant effect of *time* (F_(6,112)_ = 2; *P* = 0.16) or of *genotype* (F_(1,18)_ = 1; *P* = 0.38), and no *time* x *genotype* interaction (F_(6,112)_ = 1; *P* = 0.21; Fig. 3H). Yohimbine-induced reinstatement did not differ between genotypes (*P* = 0.99; Student's *t*-test; Fig. 3H, *inset*). Total inactive-lever presses during extinction and reinstatement were very low, did not differ between genotypes, and did not change over time (*P*>0.1; Figs. 3F, G, and H).

### Chronic ICV administration of NEI does not affect body weight or food intake

*Pmch* ^−/−^ rats lack the three neuropeptides derived from the *Pmch* precursor, NGE, NEI and MCH [4], [8]. To date, NGE does not appear to have a biological function, whereas NEI has been implicated in dopamine system modulation in several brain regions [45]. Therefore, we administered NEI or aCSF ICV in *pmch* ^+/+^ and *pmch* ^−/−^ rats for 26 days using osmotic minipumps and measured body weight and food intake.

**Figure 4:**
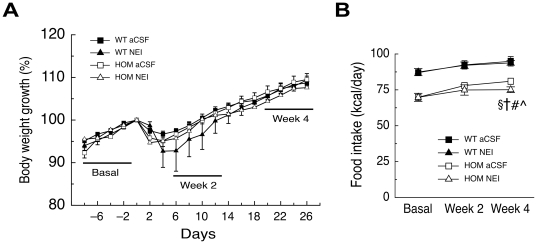
Chronic ICV administration of NEI in *pmch* ^−/−^ rats does not affect body weight or food intake. (**A**) Changes in body weight growth during 26-day ICV aCSF infusion in *pmch* ^+/+^ (n = 10) and *pmch* ^−/−^ (n = 6) rats or 26-day ICV NEI infusion in *pmch* ^+/+^ (n = 3) and *pmch* ^−/−^ (n = 4) rats (**A**). Rat body weight during operation (day 0) was set at 100%. (**B**) Average food intake (expressed as kcal/day) during aCSF or NEI infusion in *pmch* ^+/+^ and *pmch* ^−/−^ rats before minipump implantation (basal week), or during administration (week 2 or 4 as indicated in A); §, *P*<0.005, WT aCSF vs. HOM aCSF; †, *P*<0.05, WT NEI vs. HOM aCSF; #, *P*<0.001, WT aCSF vs. HOM NEI; ¯, *P*<0.005, WT NEI vs. HOM NEI, repeated-measures ANOVA). Data are shown as mean ± S.E.M.

Administration of NEI did not affect body weight growth in either *pmch* ^+/+^ or *pmch* ^−/−^ rats as compared to *pmch* ^+/+^ or *pmch* ^−/−^ rats with aCSF administration, as statistical analysis for body weight growth revealed a significant effect of *time* (F_(4,81)_ = 113; *P*<0.001; Fig. 4A). However, neither the effect of *genotype* or *treatment*, nor the interaction between *time* and *genotype*, or *treatment*, nor the triple interaction between *genotype*, *time*, and *treatment* was significant (*P *>0.1). Average home-cage food intake, as measured during 7 days before osmotic pump implantation (basal week) or during days 7–13 (week 2) or days 20–26 (week 4) after osmotic pump implantation, was also not affected by NEI administration in either *pmch* ^+/+^ or *pmch* ^−/−^ rats, as statistical analysis for food intake revealed a significant effect of *time* (F_(2,38)_ = 21; *P*<0.001) and of *genotype* (F_(3,19)_ = 17; *P*<0.001; Fig. 4B). However, neither the effect of *treatment*, nor the interaction between *time* and *genotype*, or *treatment*, nor the triple interaction between *genotype*, *time*, and *treatment* was significant (*P *>0.1; Fig. 4B). Average water intake, as measured during the basal week, week 2, or week 4, was not affected by NEI administration in either *pmch* ^+/+^ or *pmch* ^−/−^ rats (data not shown).

### MCH administration to the AcbSh of pmch^−/−^ rats rescues feeding behavior to wild-type levels

Acute administration of MCH (1* µ*g/side) or a MCH1R-agonist (5* µ*g/rat) to the AcbSh of wild-type rats increases food intake [15], [21]. Therefore we investigated whether bilateral administration of MCH (1* µ*g [419pmol] per side) to the AcbSh of *pmch* ^−/−^ rats would elevate home-cage feeding behavior to wild-type levels during the first 4 hr of the dark phase. We also tested if bilateral co-administration of NEI and NGE (1* µ*g each [691 and 509pmol, respectively] per side) to the AcbSh of nondeprived *pmch* ^−/−^ rats would affect home-cage feeding behavior.

**Figure 5:**
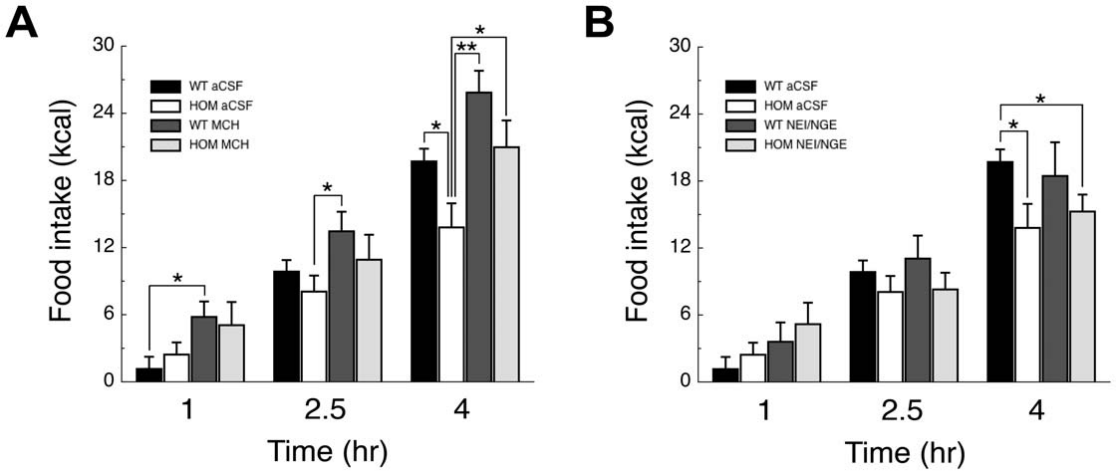
Acute AcbSh administration of MCH elevates home-cage feeding behavior in *pmch* ^−/−^ rats towards wild-type levels. (**A**) Food intake (expressed in kcal) measured 1, 2.5, and 4 hr after aCSF or MCH administration (1* µ*g/side) in the AcbSh of *pmch* ^+/+^ and *pmch* ^−/−^ rats at the beginning of the dark phase. (**B**) Food intake measured 1, 2.5, and 4 hr after aCSF administration or co-administration of NEI and NGE (1* µ*g each/side) in the AcbSh of *pmch* ^+/+^ and *pmch* ^−/−^ rats at the beginning of the dark phase (n = 6–8 per group). *P<0.05, **P<0.005, Student's *t*-test. Data are shown as mean ± S.E.M.

Statistical analyses revealed a significant effect of *time* (F_(2,72)_ = 174; *P*<0.001), of *treatment* (F_(2,36)_ = 5; *P*<0.05) and a *time* x *genotype* interaction (F_(2,72)_ = 6; *P*<0.01; Figs. 5A and B). In addition, both the effect of *genotype* and the interaction between *time* and *treatment* showed a trend (*P* = 0.09 and *P* = 0.08, respectively), while no significant effect was observed for the interaction between *genotype* and *treatment* (*P *>0.1) or the triple interaction between *genotype*, *time*, and *treatment* (*P *>0.1). *Post hoc* analysis revealed significant effects of MCH-treatment as compared to aCSF-treatment (*P*<0.05), but no significant effect for NEI/NGE-treatment (*P *>0.1). Within time-point analysis revealed increased food intake in MCH-treated *pmch* ^+/+^ rats as compared to aCSF-treated *pmch* ^+/+^ rats after 1 hr and 4 hr (*P*<0.05, Student's *t*-test; Fig. 5A), decreased food intake in aCSF-treated *pmch* ^−/−^ rats as compared to aCSF-treated *pmch* ^+/+^ rats after 4 hr (*P*<0.05, Student's *t*-test; Figs. 5A and B), decreased food intake in aCSF-treated *pmch* ^−/−^ rats as compared to MCH-treated *pmch* ^+/+^ rats after 2.5 hr and 4 h (*P*<0.05 and P<0.005, respectively, Student's *t*-test; Fig. 5A), and decreased food intake in NEI/NGE-treated *pmch* ^−/−^ rats as compared to aCSF-treated *pmch* ^+/+^ rats after 4 hr (*P*<0.05, Student's *t*-test; Fig. 5B). However, food intake in MCH-treated *pmch* ^−/−^ rats did not differ significantly from aCSF-treated *pmch* ^+/+^ rats after 4 hr (106±12% in *pmch* ^−/−^ rats; *P* = 0.68, Student's *t*-test; Fig. 5A). After 22 hr, food intake in aCSF-treated *pmch* ^−/−^ rats was decreased as compared to aCSF-treated *pmch* ^+/+^ rats (89±1% in *pmch* ^−/−^ rats; *P*<0.05, Student's *t*-test; data not shown).

Finally, after 22 hr food intake of MCH-treated rats did not differ significantly from food intake of aCSF-treated rats with the same genotype (data not shown).

### Chronic loss of Pmch in the rat affects the dopamine system

Several studies in *Pmch*- or *Mch1r*-deficient mice have shown increased dopamine release and increased dopamine receptor levels [28], [29], [30], [31], suggesting that the dopamine system in the NAc of *pmch* ^−/−^ rats might also be affected.

**Figure 6:**
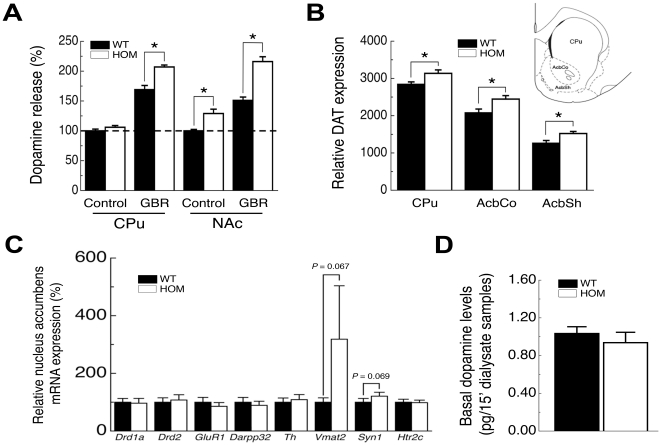
Striatal dopamine system dynamics in *pmch* ^−/−^ rats. (**A**) Electrically evoked control dopamine release is similar between genotypes in the CPu, but is increased in the NAc of *pmch* ^−/−^ rats compared to *pmch* ^+/+^ rats. Electrically evoked dopamine release in both the CPu and NAc of *pmch* ^−/−^ rats is increased compared to *pmch* ^+/+^ rats after treatment with GBR12909, a highly specific dopamine transporter (DAT) inhibitor. Data is shown in comparison to wild-type control release as 100% (dotted line; *P<0.005, Students' *t*-test; n = 4 per group). (**B**) DAT expression is increased in the CPu, AcbCo, and the AcbSh of *pmch* ^−/−^ rats compared to *pmch* ^+/+^ rats. Brain areas are indicated on an atlas section from Paxinos and Watson [72] (*P<0.05, Students' *t*-test; n = 8–11 per group). (**C**) Relative gene expression of a subset of genes involved in dopaminergic storage capacity and dopamine signaling in the NAc of adult *pmch* ^+/+^ and *pmch* ^−/−^ rats revealed an increased trend of *Vmat2* and *Syn1* in *pmch* ^−/−^ rats compared to *pmch* ^+/+^ rats (*P* = 0.067 and *P* = 0.069 by Students' *t*-test, respectively; n = 8–9 per group). (**D**) Average basal extracellular AcbSh dopamine levels did not differ between fasting *pmch* ^+/+^ and *pmch* ^−/−^ rats (n = 7 per group). Data are shown as mean ± S.E.M.

First we investigated if NAc dopamine release differs between genotypes using challenged conditions in neurochemical experiments (i.e. using electric stimulation), revealing that electrically evoked dopamine release *ex vivo* was elevated in acute coronal NAc brain slice preparations from untreated *ad libitum*-fed *pmch* ^−/−^ rats compared to untreated *ad libitum*-fed *pmch* ^+/+^ rats (Fig. 6A). Moreover, sample treatment with GBR12909, a highly specific dopamine transporter (DAT) inhibitor, increased the difference in evoked NAc dopamine release even further (129±7% at basal levels vs. 144±8% with GBR12909 treatment as compared to *pmch* ^+/+^ rats; Fig. 6A). Statistical analysis revealed an effect of *genotype* (F_(1,44)_ = 52; *P*<0.001), *treatment* (F_(1,44)_ = 112; *P*<0.001), and a *genotype* x *treatment* interaction (F_(1,44)_ = 7; *P*<0.05; Fig. 6A). This suggested that NAc DAT protein levels were increased in *pmch* ^−/−^ rats, as was recently shown for *pmch* ^−/−^ mice [29].

Radioactive ligand-binding analysis revealed that NAc DAT protein expression was indeed increased in *pmch* ^−/−^ rats, both in the nucleus accumbens core (AcbCo; 118±6%) and in the AcbSh (123±5%; Fig. 6B). Similar results were observed for the caudate putamen (CPu) in *pmch* ^−/−^ rats (Figs. 6A and B), whereas *pmch* ^−/−^ mice did not show a difference in CPu DAT levels [29].

As *ex vivo* electrically evoked dopamine release was increased in the NAc of *pmch* ^−/−^ rats, we studied relative gene expression of a subset of genes, involved in dopaminergic storage capacity or signaling, in the NAc of adult *pmch* ^+/+^ and *pmch* ^−/−^ rats. Relative expression of D_1_R (*Drd1a*), D_2_R (*Drd2*), GluR1 (*Gria1*), DARPP32 (*Darpp32*), tyrosine hydroxylase (*Th*), and 5-hydroxytryptamine (serotonin) receptor 2c (*Htr2c*) was unchanged between genotypes (Fig. 6C). However, both relative expression of VMAT2 (*Vmat2*) and Synapsin1 (*Syn1*) showed an increased trend in *pmch* ^−/−^ rats compared to *pmch* ^+/+^ rats (*P* = 0.067 and *P* = 0.069 by Students' *t*-test, respectively; Fig. 6C).

*Vmat2* is responsible for transmitter loading of synaptic vesicles [46], is preferentially expressed in the CNS [47], [48], and increased expression of *Vmat2* increases NAc dopaminergic storage capacity [49]. The G protein subunits, G_o2_ and G_αo2_, are involved in the negative regulation of VMAT2 activity [50], whereas the MCH-MCH1R system signals via G_i/o_ [38], [51], [52], [53], [54]. Synapsin 1 controls the fraction of synaptic vesicles available for release [55], and elevated *Syn1* expression can thereby increase the efficiency of dopamine release observed in *pmch* ^−/−^ rats in this study. Therefore, our data suggest that loss of the negative modulation of MCH-mediated signaling via G_i/o_ might affect vesicle dynamics in the presynaptic terminal.

Finally, we investigated if these presynaptic adaptations result in elevated extracellular dopamine levels *in vivo*. However, basal *in vivo* extracellular AcbSh dopamine levels measured using classical microdialysis in fasting rats did not differ between genotypes (Fig. 6D).

## Discussion

Results from the present study confirm an important role for MCH-mediated signaling in the positive control of appetitive behavior. Chronic loss of *Pmch* changed meal structure dynamics during rat development and adulthood, and decreased HF food-reinforced operant responding in adult rats. In addition, chronic ICV administration of NEI or acute co-administration of NEI and NGE in the AcbSh of *pmch* ^−/−^ rats did not affect body weight regulation, whereas acute administration of MCH in the AcbSh of *pmch* ^−/−^ rats elevated home-cage food intake towards wild-type levels. Finally, using biochemical and molecular assays we show that chronic loss of *Pmch* affects striatal dopamine system dynamics.

A robust reduction in meal size in *pmch* ^−/−^ rats during development and adulthood appears consistent with the finding that pharmacological blockade of MCH1R in wild-type DIO-rats decreased home-cage meal size but not meal frequency [56]. Moreover, the study by Kowalski *et al*. suggests that a reduced meal size is a direct effect of absent MCH-mediated signaling, resulting in the hypophagia observed in *pmch* ^−/−^ rats (this study and [8]) or after acute MCH1R-blockade in wild-type DIO rats [56]. However, loss of *Pmch* in the rat also affects energy expenditure [8]. Thus, a more detailed longitudinal metabolic analysis is necessary to identify additional parameters that might be responsible for changed body weight dynamics during chronic loss of *Pmch* in the rat.

MCH immunoreactive fibers innervate nucleus of the solitary tract (NTS) regions [57]. Therefore, *Pmch*-deficiency potentially removes the inhibitory effect of MCH on glutamate transmission between primary vagal afferents and NTS neurons [58], decreasing meal size and caloric intake. However, 4^th^ ventricle MCH injections had no effect on caloric intake [58]. Thus, it is unlikely that a reduced meal size results from a lack of MCH signaling at the level of the brainstem. However, we cannot exclude that satiety factors integrated outside the brain stem are affected in our rat model. This leaves the arcuate nucleus, paraventricular nucleus, dorsomedial nucleus, and the AcbSh as primary effecter sites of MCH function [15], [21], [58], [59]. Finally, changes in ‘natural’ and ‘unnatural’ reward-related neurochemistry and behavior observed here and in other studies [21], [28], [29], [30], [31], and the intensive crosstalk between hypothalamic and striatal brain regions indicate that the AcbSh is a likely converging site to exert the orexigenic action of MCH.

As overeating of palatable foods is driven by hedonic factors, the blunted overeating in *pmch* ^−/−^ rats as compared to *pmch* ^+/+^ rats when newly presented with an HF diet suggest a dysregulated mesolimbic regulation of feeding behavior. In addition, no difference in hypophagia was observed when rats were switched from an HF diet to a SHP diet, indicating that a potential mesolimbic functional dysregulation in *pmch* ^−/−^ rats is only observed under acute reward stimulatory conditions. *Pmch* ^−/−^ mice, bred on a C57BL6 background, demonstrated no acute overfeeding during a similar paradigm but, surprisingly, also displayed no hypophagia when fed *ad libitum* [29].

*Pmch* ^−/−^ rats showed decreased HF food-reinforced operant responding during FR1 sessions. Our observations align with recent findings that acute MCH1R-blockade reduces HF food-reinforced operant responding, supporting the hypothesis that MCH1R-antagonism accelerates satiety mechanisms after the initiation of food intake, as speculated by Nair *et al*. [27]. Moreover, changes in satiety mechanisms do not appear to be short-term as for instance with Cholecystokinin, which acts within 15 minutes [60], but it seems to be a more gradual effect. This notion is supported by our observations that loss of *Pmch* had no clear effect on pellet intake during the first 30 minutes of self-administration during FR1 sessions or on food intake during the first 2.5 hours of the dark phase of *ad libitum*-fed aCSF-treated *pmch* ^+/+^ and *pmch* ^−/−^ rats in the AcbSh infusion experiment. In sum, our data suggest that MCH-mediated signaling is not crucial for the initiation of feeding, but it rather amplifies food intake or decreases satiety after the initiation of feeding.

*Pmch* ^−/−^ rats demonstrated decreased breakpoint values during PR sessions. The breakpoint value can be seen as a measure for the rewarding value of the reinforcer [61], and breakpoints were found to decrease with decreasing nutritional value of a food reward whereas they increase during food deprivation [61], [62]. This indicates that 45% HF pellets have a decreased rewarding value in *pmch* ^−/−^ rats. Finally, chronic loss of *Pmch* did not robustly affect cue-, pellet-, or yohimbine-induced reinstatement of food seeking. These findings complement recent observations that acute blockade of MCH1R-mediated signaling plays a minimal role in pellet-priming, cue-, or yohimbine-induced reinstatement [27].

*Pmch* encodes for NGE, NEI, and MCH [4], and our rat model lacks all three neuropeptides [8]. Here we show that chronic ICV administration of NEI had no effect on body weight regulation in either *pmch* ^+/+^ or *pmch* ^−/−^ rats. This observation strengthens earlier findings that acute ICV NEI administration did not affect food intake in wild-type rats, nor did it affect the orexigenic effect of ICV MCH administration [11]. Furthermore, acute co-administration of 1* µ*g NEI and 1* µ*g NGE in the AcbSh of *pmch* ^+/+^ and *pmch* ^−/−^ rats also did not affect home-cage feeding behavior. In sum, our results indicate that loss of either NEI- or NGE-mediated signaling does not contribute to the aberrant feeding behavior in *pmch* ^−/−^ rats, implicating an important role for MCH.

Acute administration of MCH or a MCH-analog to the AcbSh of wild-type rats increased food intake [15], [21], suggesting that administration of MCH to the AcbSh of *pmch* ^−/−^ rats could elevate food intake towards wild-type levels. First, acute AcbSh administration of 1* µ*g MCH elevated, albeit not significantly, food intake in wild-type rats. Furthermore, we show that acute administration of 1* µ*g MCH to the AcbSh of *pmch* ^−/−^ rats indeed elevated home-cage feeding behavior towards wild-type levels. These observations indicate that administration of 1* µ*g MCH in the AcbSh of *pmch* ^−/−^ rats is sufficient to temporarily elevate food intake towards wild-type levels. Finally, it confirms the important role for the AcbSh in MCH-mediated control of feeding behavior.

Several studies have shown that loss of MCH-mediated signaling affects striatal dopamine system dynamics [28], [29], [30], [31]. Although a direct correlation between dysregulated striatal dopamine function and hypophagia after loss of MCH-mediated signaling has not yet been demonstrated, it is tempting to speculate that there is a link between the two observations. Here we observed that chronic loss of *Pmch* in the rat resulted in increased presynaptic dopaminergic release capacity, increased DAT levels in the NAc of *pmch* ^−/−^ rats, and an increased trend in NAc gene expression of *Syn1* and *Vmat2*. Finally, basal extracellular AcbSh dopamine levels were not changed in fasting rats, indicating that under non-stimulated conditions, *pmch* ^−/−^ rats do not appear to have an increased dopamine tone. However, in our set-up, increased AcbSh DAT levels might potentially mask an increased dopamine tone. Although our data clearly indicate that the striatal dopamine system is affected in the rat after chronic loss of *Pmch*, the physiological relevance of these changes remain to be studied in more detail.

*Pmch* ^−/−^ rats are hypophagic [8], but the exact mechanism behind the hypophagia is still unknown. Recently it has been shown that MCH administration to the AcbSh of rats affects the phosphorylation state of GluR1^ser845^, reduces surface expression of GluR1-containing AMPA receptors (AMPARs), decreases amplitude of AMPAR-mediated synaptic events, suppresses action potential firing MSNs through K^+^-channel activation, and reduces neuronal cell firing in freely moving rats [21], [38]. Therefore, it is possible that loss of MCH-mediated signaling affects striatal glutamatergic signaling and dopamine function, but that the latter effect is not causal to the observed hypophagia in *Pmch*-deficient rodents. However, this has to be studied in more detail. Moreover, in addition to the direct effects on AcbSh neuronal excitability, MCH might also indirectly influence neuronal excitability of other brain areas, such as the medial ventral pallidum and the ventral tegmental area, via its effects on the AcbSh [38], [39]. Finally, loss of MCH-mediated signaling in brain areas such as the paraventricular nucleus and dorsomedial nucleus can also contribute to the phenotypes observed in this study, and therefore also remain to be studied in more detail.

An alternative to the conclusion that chronic loss of *Pmch* directly influences motivational aspects of feeding is that *Pmch* deletion could result in a defect in hypothalamic brain development resulting in hypophagia and a changed metabolic rate. This argument is formally difficult to exclude; however, no gross neuroanatomical defects were observed in brain sections from rats lacking *Pmch*. Furthermore, direct evidence for a pharmacological etiology is provided by the modulation of feeding behavior by acute or chronic administration of MCH, MCH-analogues, or MCH1R-antagonists, as described above.

MCH-mediated signaling between the LHA and the NAc has been implicated in communicating the hedonic, or rewarding aspects of feeding [63]. We here show that chronic loss of *Pmch* affects motivational aspects to obtain food and thus provides a crucial signal with which hypothalamic neural circuits controlling energy balance guide frontal brain areas to shift motivation towards food. Without MCH-mediated signaling, motivation away from food appears to prevail. In addition, others and we show that chronic loss of MCH-mediated signaling affects striatal dopamine function [28], [29], [30], [31], an ability that is shared by other LHA factors [64], [65]. Incorrect control of food intake is one of the hallmarks for developing or maintaining obesity. Therefore, the development of an anti-obesity treatment based on central MCH1R-antagonism could potentially be a way to reverse obesity by changing the motivational aspects of feeding behavior.

## Materials and Methods

### Ethics statement

The Animal Care Committees of the Royal Netherlands Academy of Arts and Sciences, the University Medical Center Utrecht, and the Free University of Amsterdam approved all animal procedures according to Dutch legal ethical guidelines.

### Animals

*Pmch* ^+/+^ and *pmch* ^−/−^ rats, on a Wistar background [8], were socially housed (2 per cage) unless noted otherwise in a temperature- and humidity-controlled room (21±2°C and 60% relative humidity) under a 12 hr light-dark cycle (lights on at 6 AM). Standard diet (semi-high protein [SHP]: RM3, 27% protein, 12% fat, 62% carbohydrate, 3.33 kcal/g AFE, SDS, Witham, United Kingdom) and water was available *ad libitum* unless noted otherwise. Rats used for the self-administration experiments were housed under a reversed 12 hr light-dark cycle with standard diet (lights on at 6 PM; Teklan Global 2016, 22% protein, 12% fat, 66% carbohydrate, 3.0 kcal/g AFE, Harlan, Horst, The Netherlands) and water available *ad libitum*. Rats used in the AcbSh infusion experiments were on a 12 hr light-dark cycle (lights on at 5 AM) and also fed Teklan Global 2016. Only male rats were used in the present study.

### Genotyping

Rats were genotyped using the KASPar SNP Genotyping System (KBiosciences, Hoddesdon, UK) as described before [8]. Rats were genotyped around postnatal day (PND) 21, and genotypes were reconfirmed after experimental procedures were completed.

### Meal structure analysis

Rats were placed individually into monitoring cages, and allowed to acclimatize for 2 days. Body weight, food and water intake were measured daily. Water and SHP diet were available *ad libitum*. Meal structures were determined from 2 consecutive days during each experimental time point using data collected by Scales (Department Biomedical Engineering, UMC Utrecht, the Netherlands). This program records the weight of food hoppers in the home cage automatically every 12 seconds, as well as the amount of licks from water bottles. A meal was defined as an episode of food intake with a minimal consumption of 1 kilocalorie (0.3 g of chow). Two consecutive meals were separated by a minimal interval of 5 minutes [66], [67]. Data analysis using a longer minimal interval did not result in appreciable changes of the results. Parameters (food intake, total meal duration, average meal duration, meal frequency, average intermeal interval, average meal size, rate of eating, and satiety ratio) were measured at PNDs 40, 58, 70, 84, and 98. If not in the monitoring cages, rats were housed together (2 per cage) in their home cage. The intermeal interval was defined as the interval between the last response of a meal to the first response of the next meal. Rates of eating were calculated by dividing each meal size by its respective duration. Finally, the satiety ratio, an index of the non-eating time (i.e., satiety) produced by each gram of food consumed, was calculated as the average intermeal interval divided by the average meal size [68].

### Acute hyperphagia assay

Adult rats (≥12 weeks old) were housed individually and after 3 days of acclimatizing, food intake was measured for 5 consecutive days. On day 6, rats received high-fat (HF) diet (45%-AFE, 20%kcal protein, 45%kcal fat, 35%kcal carbohydrates, 4.54 kcal/g AFE, SDS, Witham, United Kingdom) for 4 consecutive days. To measure acute hypophagia, rats were grown up on HF diet after weaning, and were newly presented with standard SHP diet at an adult age (≥12 weeks old) with the same set-up as described above.

### Drugs

Yohimbine-HCl (Sigma-Aldrich, Zwijndrecht, The Netherlands) was dissolved in sterile saline solution (NaCl, 0.9%). MCH (Bachem), NEI (Bachem), and NGE (Bachem) were dissolved in artificial CSF (aCSF) immediately before use.

### Food self-administration

The food self-administration experiments were conducted in standard, ventilated, and sound-attenuating operant conditioning test chambers (Med Associates Inc.). The chambers were fitted with a dim red house light and two small levers separated 15 cm from each other. Water was available *ad libitum*. A pellet receptacle was placed in between the levers. One lever was designated as ‘active’; lever pressing on this lever resulted in the delivery of one 45 mg pellet containing 45% fat and 34% carbohydrate (F05879, 5.85 kcal/g AFE; Bioserv, San Diego, CA). At the same time a cue light above the active lever was turned on for 5 sec and 6 sound clicks were produced during 3 sec (compound cue). Lever presses on the inactive lever were monitored, but were without consequences. A 15 sec time-out period immediately followed each pellet delivery during which lever pressing was without consequences. A computer interfaced to the chambers was used for equipment operation and data collection. Med PC IV software (Med Associates Inc.) was used to analyze data.

### Acquisition (FR1) and progressive ratio (PR) schedules

Adult (≥12 weeks old) *pmch* ^+/+^ rats weighing between 330–350 g and *pmch* ^−/−^ rats weighing between 280–300 g at the beginning of the experiment were used. Home-cage food intake was measured during 4 days before the start of the experiment, and all rats received approximately 80% of their regular food intake during the self-administration paradigm. Body weight and food intake were measured daily during the course of the study. Acquisition phase sessions (3 hr duration, with cues: cue light on for 5 sec, 6 sound clicks during 3 sec) commenced after 7 days of acclimation to the animal facility and were performed between 10 AM and 1 PM. Rats were allowed to self-administer pellets during 12 daily sessions on an intermittent (1 day) fixed ratio 1 (FR1) schedule of reinforcement. After the FR1 schedule, rats were allowed to self-administer pellets on a progressive ratio (PR) schedule during 4 intermittent (1 day) sessions (3 hr duration, with cues: cue light on for 5 sec, 6 sound clicks during 3 sec). The successive increase in number of lever presses required to obtain a pellet delivery was calculated by the following equation: Response ratio = (5e^(0.2*reward number)^)−5, rounded to the nearest integer [44]. This equation produced the following sequence of required lever presses: 1, 2, 4, 6, 9, 12, 15, 20, 35, 40, 50, 62, 77 etcetera. The final ratio attained was defined as the animal's breakpoint.

### Extinction phases and reinstatement

After the PR sessions, rats were allowed to self-administer pellets at an FR1 schedule for 4 intermittent (1 day) sessions (3 hr duration, with cues: cue light on for 5 sec, 6 sound clicks during 3 sec). Rats then entered an extinction phase of 22 consecutive daily sessions (1 hr duration, no reward, no cues). After all rats showed stable extinction values (<10 active responses, 5 consecutive sessions), rats were tested for cue-induced relapse (1 hr duration, no reward, 1 cue series at start of session and the ability to respond for the compound cue on an FR1 schedule). Rats then entered an extinction phase of 9 consecutive daily sessions during which responding for the cues was extinguished (1 hr duration, no reward, with cues). After all rats showed stable extinction values (<10 active responses, 5 consecutive sessions) rats were tested for pellet-induced relapse (1 hr duration, no reward, 1 pellet at start of session, with cues). Following 14 additional extinction sessions rats were tested for yohimbine-induced relapse (1 hr duration, no reward, yohimbine [2 mg/kg, 1 ml/kg, intraperitoneal, 30 min prior to start of session], with cues). Yohimbine is an *α*_2_-adrenergic receptor antagonist that induces stress-like responses [69].

### Chronic intracerebroventricular NEI administration

Body weight, food-, and water intake of individually housed rats were measured for 8 days (PNDs 86–94). At PND 94, rats were anesthetized with isoflurane, and received one dose of Temgesic (0.05 mg/kg subcutaneous; Schering-Plough, Utrecht, the Netherlands). Average body weight at PND 94 was 400 g for *pmch* ^+/+^ rats and 323 g for *pmch* ^−/−^ rats. A sterile brain infusion cannula (28-gauge; Brain infusion kit 1; Alzet, Palo Alto, CA) was stereotaxically implanted into the left third ventricle. When a flat skull position was used, the stereotaxic coordinates were 0.9 mm caudal to the bregma, 1.5 mm lateral to the midline, and 3.5 mm (*pmch* ^+/+^ rats) or 3.0 mm (*pmch* ^−/−^ rats) from the surface of the skull. The cannulae were fixed to the skull with dental cement. The infusion cannula was connected to an osmotic minipump (Pump model 2002, Alzet, Palo Alto, CA) that was filled with aCSF. The osmotic pumps were placed under the dorsal skin of the neck and connected to the ICV cannula using plastic tubing (length 9 cm; i.d. 0.69 mm; o.d. 1.14 mm). At the start of the study each cannula was filled with aCSF. After distribution into matched experimental and control groups, *pmch* ^+/+^ rats and *pmch* ^−/−^ rats received a minipump filled with either aCSF (0.5* µ*l/hr) or NEI (dissolved in aCSF; 8* µ*g/rat/day). Body weight, food-, and water intake were then recorded every two days for 13 days. After 13 days, at PND 107, all rats received a new mini pump under isoflurane anesthesia. Subsequently, body weight, food-, and water intake were recorded every two days for another 13 days. At the end of all experiments, the animals were sacrificed and the position of the ventricular cannula assessed following the injection of 150* µ*l of Evans blue dye (2 mg/ml) and visual examination of brain slices. Only parameters recorded from animals with correctly positioned cannulae were included in the results. Body weight at PND 94 was set as 100%. Average food and water intake was calculated for PNDs 86–93 (basal week), PNDs 100–106 (week 2), or PNDs 114–120 (week 4).

### AcbSh surgery

Adult *pmch* ^+/+^ and *pmch* ^−/−^ rats (≥15 weeks old) received a guide cannula (Plastics One) in the AcbSh bilaterally, as described before [21]. After surgery, the rats were allowed to recover for 7 days, followed by daily manipulation and habituation to the injection procedure for 3 days.

### AcbSh microinjection procedure

AcbSh microinjection procedure was performed as described before [21]. Tubing was coated with 2% BSA solution to minimize loss of MCH to the tubing. Reagent was resuspended in aCSF immediately before use.

### AcbSh feeding behavior assay

On a test day, food was removed at 4 PM, and the rats were allowed to remain in their home cage until the drug or vehicle control was injected into the AcbSh using a full Latin square design (aCSF, 1* µ*g MCH per side, or 1* µ*g NEI and 1* µ*g NGE per side). After the injection, the rats were returned to their home cage, and 1 hr later they were allowed full access to their chow. The experiment started at the beginning of the dark cycle (5 PM), and food and water intake were measured for 1, 2.5, 4, and 22 hr after the start of the dark cycle. Each test session was separated by 3 days. No evidence of order or carryover was observed in the Latin square design.

### AcbSh histology

At the end of the experiment, rats were given an overdose of sodium-pentobarbital (200 mg/kg, intraperitoneal) and were intracardially perfused with 60 ml 4% paraformaldehyde solution. Vibratome sections (35* µ*m) were cut to determine the correct location of the guide cannulae. Only data from rats with two correctly placed cannulae were included.

### Neurochemical analysis

Adult rats (≥12 weeks old) were decapitated and the caudate putamen (CPu) and nucleus accumbens (NAc) were rapidly dissected from the coronal brain slices. Samples (0.3×0.3×2 mm) were prepared using a McIlwain tissue chopper, incubated and superfused essentially as described before [70]. Samples were washed twice with Krebs-Ringer bicarbonate medium (in mM; NaCl, 121; KCL, 1.87; KH_2_PO_4_, 1.17; MgSO_4_, 1.17; NaHCO_3_, 25; CaCl_2_, 1.22 and D−(+)-glucose, 10), followed by preincubation for 15 min in this medium in a constant atmosphere of 95% O_2_-5% CO_2_ at 37°C. After preincubation, the samples were washed rapidly with the Krebs-Ringer and incubated for 15 min in 2.5 mL of this medium containing 5* µ*Ci [^3^H]dopamine in an atmosphere of 95% O_2_-5% CO_2_ at 37°C with or without 6* µ*M GBR-12909 (dopamine reuptake inhibitor; Sigma-Aldrich, Zwijndrecht, the Netherlands). As the CPu and the NAc have a dense noradrenergic innervation, 3.6* µ*M desipramine (3-isobutyl-1-methyl-xantine [DMI]; Sigma, St. Louis, MO, USA) was added to the medium of these brain structures to prevent accumulation of [^3^H]dopamine in noradrenergic nerve terminals. After labeling, the samples were washed rapidly and transferred to a chamber of the superfusion apparatus (approximately 4 mg tissue in 0.2 mL volume) and superfused (0.2 mL/min) with medium gassed with 95% O_2_-5% CO_2_ at 37°C. In each observation, neurotransmitter release from samples of *pmch* ^+/+^ and *pmch* ^−/−^ rats was studied simultaneously. After 40 min of superfusion (*t* = 40 min), the superfusate was collected as 10-min samples. Neurotransmitter release was induced by exposing the samples to electrical biphasic block-pulses (1 Hz, 4 ms at 30 mA) for 10 min at *t* = 50. The radioactivity remaining at the end of the experiment was extracted from the tissue with 0.1N HCl. The radioactivity in superfusion fractions and tissue extracts was determined by liquid scintillation counting. The efflux of radioactivity during each collection was expressed as a percentage of the amount of radioactivity in the slices at the beginning of the respective collection period. The electrically evoked release of neurotransmitter was calculated by subtracting the spontaneous efflux of radioactivity from the total overflow of radioactivity during stimulation and the next 10 min. A linear decline from the 10-min interval before to that 20–30 min after the start of stimulation was assumed for calculation of the spontaneous efflux of radioactivity. The evoked release was expressed as percentage of the content of radioactivity of the samples at the start of the stimulation period.

### Autoradiographic DAT assay

Cryostatic coronal sections (10* µ*m) through mid-striatum from adult rats (≥12 weeks old) were preincubated (20 min, 20°C) in 50 mM Tris-HCl 120 mM NaCl (pH 7.4), then 1 hr in fresh buffer containing 10 pM RTI-55 (2200 Ci/mmol; Perkin Elmer) and 1* µ*M Citalopram and Nisoxetine (Sigma-Aldrich, Zwijndrecht, the Netherlands), with nonspecific binding defined with 100* µ*M GBR-12909 (Sigma-Aldrich, Zwijndrecht, the Netherlands). Slides were washed twice for 10 min in fresh buffer (4°C), dipped in ice-cold deionised water, airflow dried, exposed to tritium-sensitive film for 5 days with tritium standards, photodeveloped, and analyzed using NIH freeware ImageJ.

### NAc mRNA expression

Adult rats (≥12 weeks old) were sacrificed during the early afternoon. The NAc was rapidly dissected and snap-frozen in liquid nitrogen**.** Total RNA was isolated using a Trizol method and RNA quantity and quality was assessed using a Nanodrop ND-1000 spectrophotometer (Thermo-Scientific, Wilmington, DE, USA). cDNA was synthesized from 1* µ*g of total RNA using a RetroScript kit (Applied Biosystems, Nieuwerkerk a/d IJssel, NL) as described by the manufacturer, and diluted in MQ (1∶2 for *Th* and *Vmat2*; 1∶8 for all other genes). Gene expression was quantified with a 7900 HT Real-Time PCR machine (ABI Prism). Primers for *Cyclophilin, Drd1a*, *Drd2*, *GluR1*, *DARPP32*, *Th*, *Vmat2*, *Syn1*, and *Htr2c* were designed using SciTools PrimerQuest (IDT; primers shown in Table S2). Primers were optimized to amplify cDNA but not genomic DNA and to generate a single PCR product. PCR efficiency was between 80% and 120%. In general, 2* µ*l template, 10* µ*M primers, and 5* µ*l SYBRGreen Mix (Applied Biosystems) was used in a 10* µ*l PCR reaction. Thermocycler conditions comprised an initial holding stage at 50°C for 2 min followed by 95°C for 3 min followed by a PCR program consisting of 95°C for 30 sec and 60°C for 30 sec for 40 cycles. Samples were run in triplicates. To control for input, *Cyclophilin* was run on the same plate and used as a control gene. Calculations were performed by a comparative method (2.0^−ΔΔCt^), taking the efficiency of the PCR into account (1.8–2.2). All experiments were repeated twice after a new cDNA synthesis reaction. Average *pmch* ^−/−^ rat gene expression from the three experiments is expressed as a percentage of average *pmch* ^+/+^ gene expression.

### AcbSh microdialysis surgery

Rats were anesthetized using isoflurane (2.5%, 400 ml/min N_2_O, 600 ml/min O_2_). Lidocaine (10% m/v) was used for local anesthesia. The animals were fixed in a stereotaxic frame and unilaterally implanted with a stainless steel guide cannula (8 mm) aimed at the right AcbSh according to previously described procedures [49], [71]. The following coordinates were used: anteroposterior +10.2 mm; lateral −0.8 mm; dorsoventral −6.0 mm [72]. The anteroposterior coordinate is relative to the interaural line; the lateral coordinate is relative to the midline suture and the dorsoventral coordinate is relative to the skull surface. The cannula was fixed onto the skull and anchored with dental cement and stainless steel screws. The guide cannula contained an inner cannula to prevent infections and occlusions. The rats were allowed to recover from surgery for at least 7 days in Plexiglas microdialysis cages (25×25×35 cm) for the rest of the experiment. On 3 consecutive days, prior to the start of the microdialysis experiment, each rat was gently picked up and lifted above the top of the home cage in order to habituate them to handling.

### Microdialysis procedure

A detailed description of the microdialysis procedure has been published elsewhere [49]. In short, a dialysis probe (type A-I-8-02, outer diameter: 0.22 mm, 50,000 molecular-weight cut-off) was carefully inserted into the guide cannula. The probe was secured to the guide cannula using a screw. The tip of the dialysis probe protruded 2 mm below the distal end of the guide cannula. The probes had an *in vitro* recovery of 10–12% for dopamine. The inlet and outlet of the probe were connected to a swivel that allowed the rat to move freely inside the microdialysis cage. The dialysis probe was perfused at a rate of 2.0* µ*l/min with Modified Ringer solution (147 mM NaCl, 4 mM KCl, 1.1 mM CaCl_2_.2H_2_O and 1.1 mM MgCl_2_.6H_2_O, dissolved in ultra pure water, pH 7.4). The outflow was collected every 15 min in a tube containing 8* µ*l of 0.02 M formic acid and kept at −80°C until analyzed. The samples were manually injected into a high performance liquid chromatography (HPLC) system. Dopamine was separated from the remaining neurotransmitters by means of reversed phase, ion-paring liquid chromatography using an Eicompak PP-ODS column (particle size: 2* µ*m, 4.6 mm×30mm) in combination with a mobile phase (0.1 M phosphate buffer [NaH_2_PO_4_.2H_2_O : Na_2_HPO_4_.12H_2_O, ratio 25∶4], 2.0 mM sodium 1-decanesulphonate and 0.1 mM di-sodium EDTA, dissolved in ultra pure water [>18 MΩ], pH 6.0) containing 1% of methanol. The flow rate was 500* µ*l/min and the system temperature was 25°C. The concentration of dopamine was measured using electrochemical detection. The working electrode was set at +400 mV against a silver/silver-chloride reference electrode. The accuracy of measurement was within 1.3% and the detection limit was about 30fg per sample. The system was calibrated using a standard dopamine solution before each measurement. On the experimental day, food was removed 1 hr before start of the experiment and during the remainder of the experiment. A stable baseline level of dopamine (±10%) was reached at 4 hr after insertion of the probe [49], [71], followed by the collection of 3 basal samples. Average basal dopamine levels are expressed as pg dopamine in a 15 min dialysate sample.

### Microdialysis histology

At the end of the experiment, rats were given an overdose of sodium-pentobarbital (250 mg/kg, intraperitoneal) and were intracardially perfused with 60 ml 4% paraformaldehyde solution. Vibratome sections (35* µ*m) were cut to determine the correct location of the microdialysis probe.

### Data analysis

All data are expressed as mean ± S.E.M. Meal pattern characteristics (Figures 1A-I) were analyzed by repeated-measures ANOVA that included the between-subject factor of *genotype* and the within-subjects factor of *time*. If an effect was observed, the ANOVA was followed by an unpaired Students' *t*-test analysis. In addition, figs. 1B-G were also analyzed by an ANCOVA at each experimental time point with body weight as covariate. As we were interested in a parameter that only showed a *genotype* effect at PND 40, repeated measured ANCOVAs could not be used for this analysis. Acute hyperphagia and hypophagia (Figs. 2A and B) were analyzed by repeated-measures ANOVA with a special contrast to investigate if the intake on the test day (HF day1 or SHP day1) was different from the average intake of 5 preceding days (SHP days1–5 or HF days1–5). Elements of high-fat food-reinforced responding (Figs. 3B-H) were analyzed by repeated-measures ANOVA. If an effect was observed, the ANOVA was followed by an unpaired Students' *t*-test analysis. Body weight and food intake during chronic aCSF or NEI administration were analyzed by repeated-measures ANOVA with *genotype* and *treatment* as between-subject factors and the within-subjects factor of *time*, with a Bonferroni *post-hoc* correction for multiple comparisons. For the AcbSh infusions we used a mixed experimental design that included the between-subject factors of *genotype* and *treatment* and the within-subjects factor of *time*, with a Bonferroni *post-hoc* correction for multiple comparisons. Within time-point data were analyzed using an unpaired Students' *t*-test. The effect of GBR12909 on dopamine release was analyzed using a 2-way ANOVA with between-subject factors of *genotype* and *treatment*. All other data were analyzed using an unpaired Students' *t*-test. All data were analyzed using a commercially available statistical program (SPSS for Macintosh, version 16.0). The null hypothesis was rejected at the 0.05 level.

## Supporting Information

Table S1 **Statistical results for the meal structure analysis.**
(TIF)

Table S2 **Gene name, gene ID, and forward and reverse primer sequences for qPCR analysis of NAc gene expression.**
(TIF)
