# Supplementary figures and images for: Chronic Loss of Melanin-Concentrating Hormone Affects Motivational Aspects of Feeding in the Rat
Source: PLoS One. 2011 May 5;6(5):e19600. doi: 10.1371/journal.pone.0019600 (PMC3088702; doi:10.1371/journal.pone.0019600)

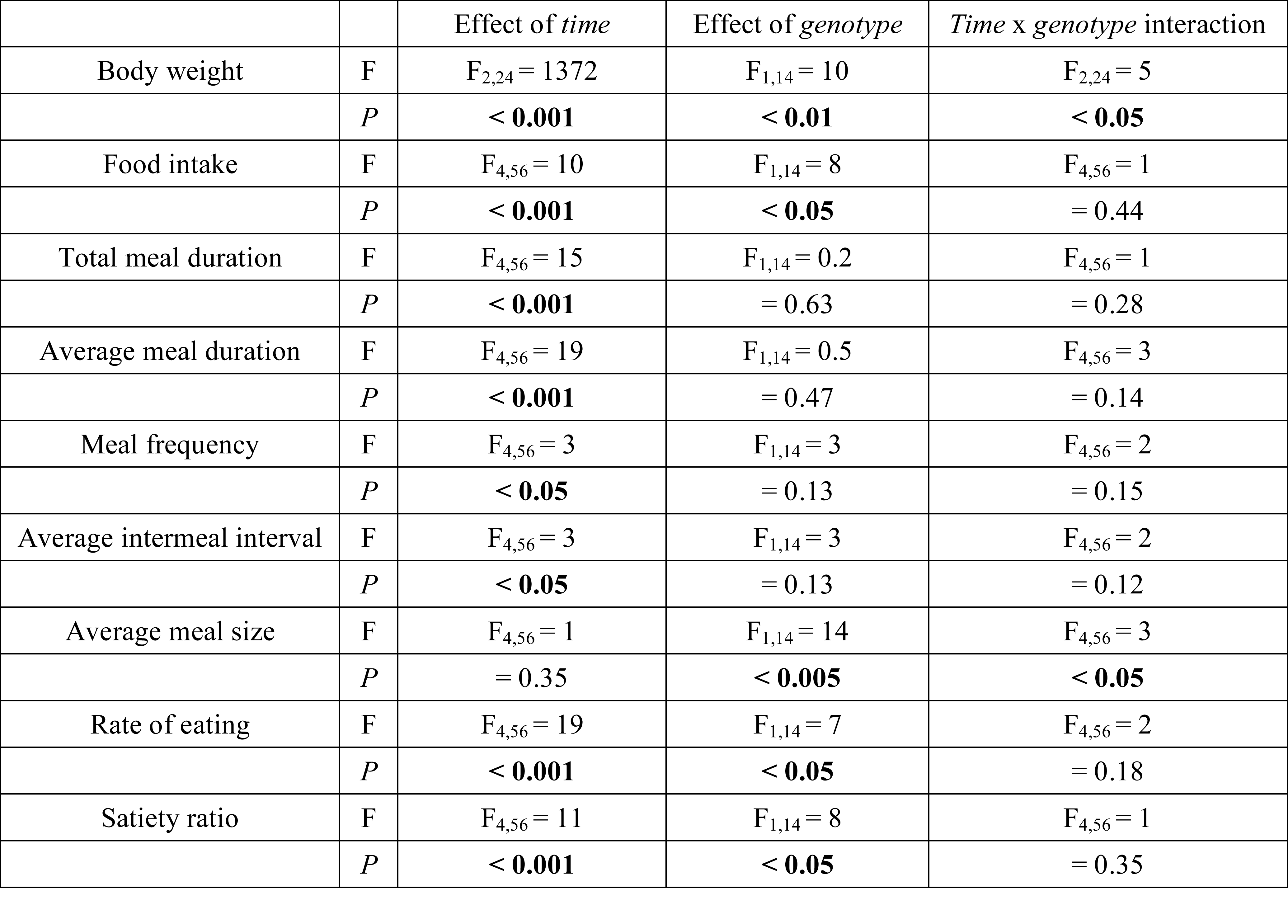

Supplement: Table S1 — Statistical results for the meal structure analysis. (TIF) [file pone.0019600.s001.tif]

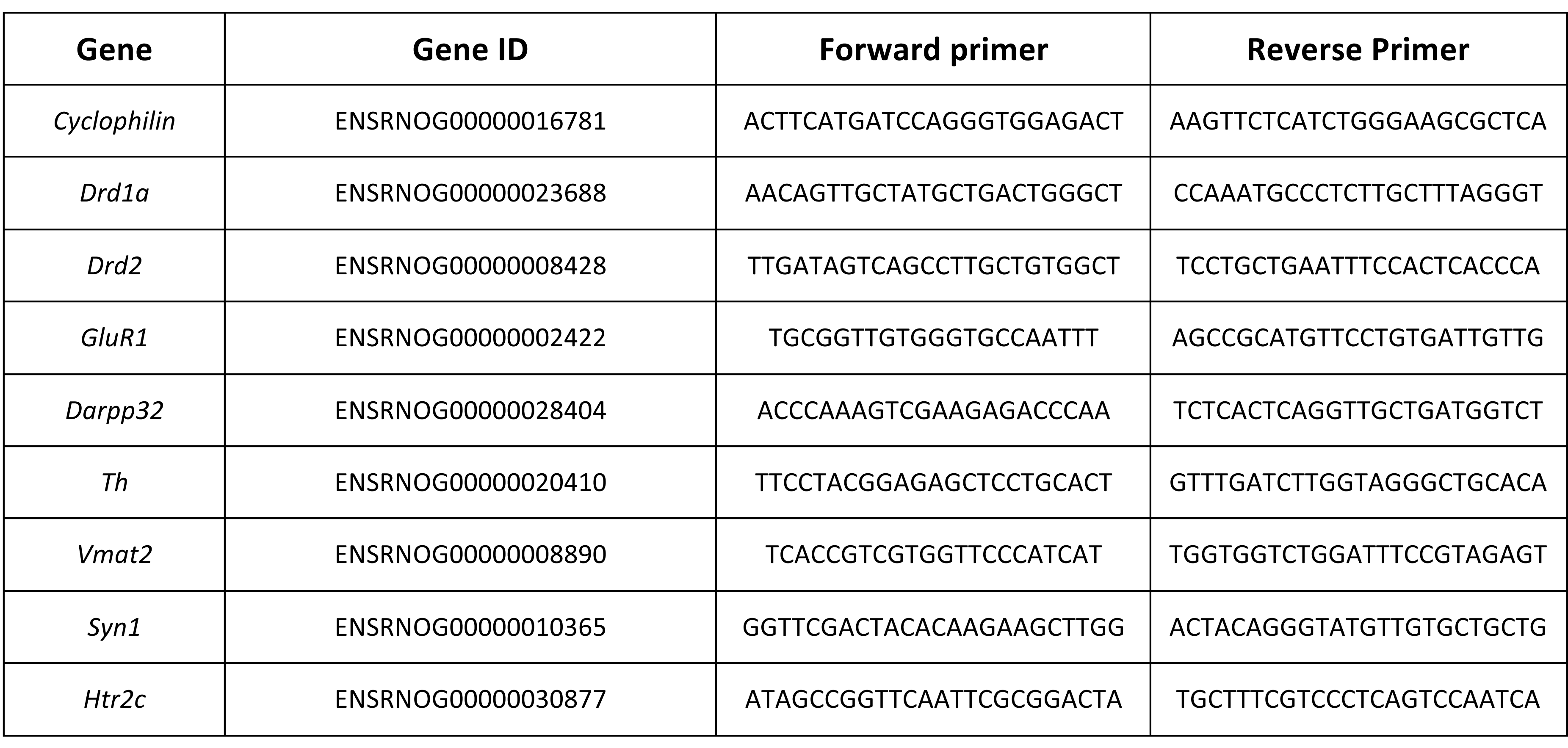

Supplement: Table S2 — Gene name, gene ID, and forward and reverse primer sequences for qPCR analysis of NAc gene expression. (TIF) [file pone.0019600.s002.tif]
